# Exploration of bioconvection flow of MHD thixotropic nanofluid past a vertical surface coexisting with both nanoparticles and gyrotactic microorganisms

**DOI:** 10.1038/s41598-021-96185-y

**Published:** 2021-08-17

**Authors:** Olubode Kolade Koriko, Nehad Ali Shah, S. Saleem, Jae Dong Chung, Adeola John Omowaye, Tosin Oreyeni

**Affiliations:** 1 Department of Mathematical Sciences, Federal University of Technology, Akure, Nigeria; 2 Department of Mechanical Engineering, Sejong University, Seoul, 05006 Republic of Korea; 3 Department of Mathematics, Lahore Leads University, Lahore, Pakistan; 4 Department of Mathematics, College of Science, King Khalid University, Abha, 61413 Saudi Arabia; 5 Department of Physical Sciences, Precious Cornerstone University, Ibadan, Nigeria

**Keywords:** Mechanical engineering, Mathematics and computing

## Abstract

This communication presents analysis of gravity-driven flow of a thixotropic fluid containing both nanoparticles and gyrotactic microorganisms along a vertical surface. To further describe the transport phenomenon, special cases of active and passive controls of nanoparticles are investigated. The governing partial differential equations of momentum, energy, nanoparticles concentration, and density of gyrotactic microorganisms equations are converted and parameterized into system of ordinary differential equations and the series solutions are obtained through Optimal Homotopy Analysis Method (OHAM). The related important parameters are tested and shown on the velocity, temperature, concentration and density of motile microorganisms profiles. It is observed that for both cases of active and passive control of nanoparticles, incremental values of thermophoretic parameters corresponds to decrease in the velocity distributions and augment the temperature distributions.

## Introduction

Rheology is explained to be the science that deals with the deformation and flow of matters, it focuses on determining the intrinsic flow behaviour of materials by studying the interrelationship between force, deformation and time. Flow curve is very significant in the course of analyzing the rheological properties of materials. With flow curve and the relationship between rheological parameters and time, materials can be classified into Newtonian and non-Newtonian fluids Bashir^1^. Non-Newtonian fluids are defined as ‘time-dependent’ fluids because, following deformation, they take a set amount of time to return to their original condition or viscous state, and the flow is heavily reliant on the viscosity. Non-Newtonian (time-dependent) fluids that exhibit shear thinning behavior are known as thixotropic fluids. Thixotropic materials exhibit complex rheological properties due to the microstructures and are usually with high concentrated solid particles Mewis and Wagner^2^. Thixotropy is a physical characteristic of solids that behave like semi-solid fluids under normal conditions but flow like liquids after being agitated. The dissociation of weak bonds between polymeric molecules, which progressively rejoin while resting, causes the changes seen in thixotropic materials. In other word, thixotropy can be referred to as a progressive decrease in viscosity over time for a constant applied shear stress, followed by gradual recovery when stress is removed Axelson^3^. Many gels and colloids are known to be thixotropic in nature, honey, synovial fluid in the joints between some bones, sand, quicksand, clays and drilling muds are some commonly found natural examples of materials that may be thixotropic Hendrickson^4^. The cytoplasm in the human blood is known to exhibit thixotropic behaviour, in the sense that the cytoplasmic matrix known as the viscous and transparent liquid part of the cytoplasm has unusual property of exhibiting a viscous flow like liquid and elastic deformations like a solid Hendrickson^4^. Due to the complexities working with this fluid, there are very few works that focus on the boundary layer flows of the fluids. Oreyeni and Omokhuale^5^ considered an analytic approach to MHD natural convection flow of thixotropic fluid subject to thermal stratification, it was reported in the study that incremental values of the thixotropic parameters correspond to increase in the velocity distributions for both cases of hypolimnion and epilimnion. Waqas et al.^6^ studied the solar radiation and joule heating in magnetohydrodynamic convective flow of thixotropic nanofluid, they found out that, increase in thixotropic parameters leads to increase in the fluid velocity. Other studies that reported boundary layer flow of thixotropic fluid can be found in^7–9^.

A variety of approaches have been proposed to improve the heat transfer efficiency of various working fluids with low thermal conductivity. The thermal conductivity of the working fluids can be increased to improve heat transfer efficiency. Nanofluids, which assist to enhance the heat transfer efficiency of fluids in industrial processes, have been introduced as a novel notion by nanotechnology. Nanofluids are a novel type of solid-liquid composite material made up of solid nanoparticles $(1{-} 100 \; \text{nm})$ suspended in a base liquid. Choi^10^. Because of their increased heat transfer efficiency in diverse thermal systems, nanofluids are employed in a wide range of engineering applications. Engine coolant, automatic transmission fluid, brake fluid, gear lubrication, engine oil, and greases are all possible applications of nanofluids^11^ Senthilraja et al. Nanomaterials are also well designed as flavor additives, or carriers for food supplement (i.e. nanoencapsulation and nanoemulsion) Weir et al.^12^. Ahmed^13^ presented the influence of slip boundary condition on Casson nanofluid flow over a stretching sheet in the presence of viscous dissipation and chemical reaction. With an increase in Brownian motion and thermophoresis parameter, the heat transfer rate is reduced, but the mass transfer rate is clearly enhanced, according to the study. Rauf et al.^14^ studied mixed convection flow of couple stress nanofluid over oscillatory stretching sheet with heat absorption/generation effects. Entropy generation in second grade magnetohydrodynamic nanofluid flow over a convectively heated stretching sheet with nonlinear thermal radiation and viscous dissipation was studied by Mondal et al.^15^, they discovered that, thermophoresis parameter acts to increase the temperature of the fluid.

The process of pattern formation observed in the aqueous suspension of motile microorganisms when they respond to certain stimuli by swimming in certain directions termed taxes is referred to as bioconvection Platt^16^. In other words, bioconvection occurs when microorganisms with a higher density than the water swim upward towards the upper surface of the water; however, when the upper surface becomes too dense due to the accumulation of microorganisms, it becomes unstable, and microorganisms swim downward to the lower layer of the water, maintaining bioconvection. Ghorai and Hill^17^ explained that gyrotaxis is swimming directed by the balance between the torque due to gravity acting on a bottom-heavy cell and the torque due to viscous forces arising from local shear flows. Raees et al.^18^ reported that bioconvection in nanofluids has great potential in Colibri micro-volumes spectrometer and also to improve the stability of nanofluids. They further revealed the application of bioconvection in the field of microbial enhanced oil recovery, which involves injection of selected microorganisms into the reservoir and by their situ multiplication they reduce the residual oil left in the reservoir after secondary recovery is exhausted. Makinde and Animasaun^19^ considered bioconvection in MHD nanofluid with nonlinear thermal radiation and quartic autocatalysis chemical reaction past an upper surface of a paraboloid of revolution. It was reported that velocity profiles and density of motile microorganisms are decreasing functions of Schmidt number for diffusing motile microorganism. Recently, Saleem et al.^20^ discussed magneto Jeffrey nanofluid bioconvection over a rotating vertical cone due to gyrotactic microorganisms. It was discovered that when the bioconvection Peclet number and bioconvection Schmidt number increase, the rescaled density of motile microorganisms decreases.

Temperature and concentration differences, respectively, are known to be driving forces for heat and mass transfer. Furthermore, both heat and mass are considered to be transferred from the region of higher concentration to the region of lower concentration. The thermal relaxation time expression was initially incorporated into Fourier’s law by Catteneo^21^. By adding the Oldroyd upper-convected derivative, Christov^22^ improved on Catteneo’s hypothesis. The Catteneo-Christov heat and mass theory is the name given to the theory currently (CCH-model). Sarojamma et al.^23^ analyzed the effects of an autocatalytic chemical reaction and a Catteneo-Christov heat flux on the dynamics of a micropolar fluid. They discovered that when the thermal relaxation parameter is set to zero, the temperature of the fluid declines and the Catteneo-Christov (CCH) heat flux model is simplified to the traditional Fourier’s Law of conduction. Rasool and Zhang^24^ considered Darcy-Forchheimer nanofluid flow manifested with Cattaneo-Christov theory of heat and mass flux over non-linearly stretching surface. It was discovered that the solutal relaxation time parameter exhibits mixed behavior in nanoparticles concentration distribution. Sandeep et al.^25^ considered free convective MHD Cattaneo-Christov flow over three different geometries with thermophoresis and Brownian motion. It was reported that thermal relaxation parameter helps to enhance the heat transfer rate but lessened the mass transfer rate.

To the best of authors’ knowledge, numerous studies have been reported on boundary layer flow of nanofluid in the presence of nanoparticles and gyrotactic microorganisms manifesting with the Catteneo-Christov heat and mass flux model (CCH) in the recent years. Furthermore, it is worth mentioning that there is no research on the comparison between the active and passive controls of nanoparticles of thixotropic fluid flow in the presence nanoparticles and gyrotactic microorganisms coexisting with Catteneo-Christov heat and mass flux model (CCH).

## Mathematical formulation

In line with boundary layer theory a steady, buoyant convective boundary layer equation of thixotropic-nanofluid is explored to meet our objectives. The model is modified to a particular extent where the mathematical formulation of the problem can be justified. The flow is assumed to be in the *x*-direction, which is taken vertically upward along the plate, and the *y*-direction is normal to it. $(T_w)$, nanoparticle concentration $(C_w)$, and motile microorganism density $(N_w)$ at the stretching surface are assumed to be constant and are considered to be greater than temperature $(T_\infty )$, nanoparticles concentration $(C_\infty )$, and motile microorganisms density $(N_\infty )$ at the free stream, respectively. The presence of nanoparticles is thought to have no influence on the direction and speed with which microorganisms swim. It is assumed that bioconvection flow occurs only in dilute nanoparticles suspensions. It is worth mentioning that the base fluid is water so that the microorganisms can survive. The active and passive controls of nanoparticles volume fraction at the boundary are both taken into account (on the solid wall). Under the foregoing assumptions with Boussinesq approximation, and following the works of Rehman et al.^26^ and Shehzad^8^, the governing equations for mass, momentum, energy, and density of gyrotactic microorganisms in two-dimensional thixotropic-nanofluid may be expressed as follows;

Continuity Equation

1 $$\begin{aligned} \frac{\partial u}{\partial x} + \frac{\partial v}{\partial y} = 0, \end{aligned}$$

Momentum Equation

2 $$\begin{aligned} u \frac{\partial u}{\partial x} + v \frac{\partial u}{\partial y} & = \frac{\mu }{\rho }\left( \frac{\partial ^2 u}{\partial y^2}\right) - \frac{6R_1}{\rho }\left( \frac{\partial u}{\partial y}\right) ^2 \frac{\partial ^2 u}{\partial y^2} +\frac{4R_2}{\rho } \left( \frac{\partial u}{\partial y}\right) \left( \frac{\partial ^2 u}{\partial y^2}\right) \left( u\frac{\partial ^2 u}{\partial x \partial y} + v \frac{\partial ^2 u}{\partial y^2}\right) \nonumber \\ & \quad +\frac{4R_2}{\rho }\left( \frac{\partial u}{\partial y}\right) ^2 \left( u\frac{\partial ^3 u}{\partial x \partial y^2} + v \frac{\partial ^3 u}{\partial y^3} +\frac{\partial u}{\partial y}\frac{\partial ^2 u}{\partial x \partial y} + \frac{\partial v}{\partial y} \frac{\partial ^2 u}{\partial y^2}\right) -\sigma \frac{B_o^2 u}{\rho }\nonumber  \\ & \quad + (1-C_{\infty })\rho _f g\beta (T-T_\infty ) - g(\rho _p - \rho _f)(C-C_\infty )-g \gamma (\rho _m - \rho _f)(N-N_\infty ), \end{aligned}$$

Energy equation of nanoparticles

3 $$\begin{aligned}  u\frac{\partial T}{\partial x} + v\frac{\partial T}{\partial y} & = \frac{\kappa }{\rho C_p}\frac{\partial ^2 T}{\partial y^2} + \tau \left( D_B \frac{\partial T}{\partial y}\frac{\partial C}{\partial y} + \frac{D_T}{T_\infty }\left( \frac{\partial T}{\partial y}\right) ^2\right) \nonumber \\ & \quad + \lambda _1\left( u^2 \frac{\partial ^2 T}{\partial x^2} + v^2 \frac{\partial ^2 T}{\partial y^2} + 2uv \frac{\partial ^2 T}{\partial y \partial x} + u \frac{\partial u}{\partial x} \frac{\partial T}{\partial x} + u \frac{\partial v}{\partial x} \frac{\partial T}{\partial y} + v \frac{\partial u}{\partial y} \frac{\partial T}{\partial x} + v \frac{\partial v}{\partial y}\frac{\partial T}{\partial y}\right) , \end{aligned}$$

Concentration equation of nanoparticles

4 $$\begin{aligned}  u\frac{\partial C}{\partial x} + v\frac{\partial C}{\partial y} & = D_B\frac{\partial ^2 C}{\partial y^2} + \frac{D_T}{T_\infty }\frac{\partial ^2 T}{\partial y^2}\nonumber \\ & \quad + \lambda _2\left( u^2 \frac{\partial ^2 C}{\partial x^2} + v^2 \frac{\partial ^2 C}{\partial y^2} + 2uv \frac{\partial ^2 C}{\partial y \partial x} + u \frac{\partial u}{\partial x} \frac{\partial C}{\partial x} + u \frac{\partial v}{\partial x} \frac{\partial C}{\partial y} + v \frac{\partial u}{\partial y} \frac{\partial C}{\partial x} + v \frac{\partial v}{\partial y}\frac{\partial C}{\partial y}\right) , \end{aligned}$$

Density of gyrotactic microorganisms equation

5 $$\begin{aligned} u\frac{\partial N}{\partial x} + v\frac{\partial N}{\partial y} + \frac{bW_c}{(C_w - C_{\infty }}\left[ \frac{\partial }{\partial y}\left( N \frac{\partial C}{\partial y}\right) \right] = D_m \frac{\partial ^2 N}{\partial y^2} \end{aligned}$$

The equations above are subjected to the following two sets of boundary conditions:

(i) The actively controlled nanofluid model
  6 $$\begin{aligned}&u = ax, \;\;\; v = 0, \;\;\; T=T_w, \;\;\; C = C_w, \;\;\; N = N_w \;\;\;\;\ at \;\;\; y =0,\nonumber \\&u \rightarrow 0, \;\;\; T\rightarrow T_\infty , \;\;\; C \rightarrow C_\infty , \;\;\; N \rightarrow N_\infty \;\;\;\;\ as \;\;\; y \rightarrow \infty \end{aligned}$$
(ii) The passively controlled nanofluid model
  7 $$\begin{aligned}&u = ax, \;\;\; v = 0, \;\;\; T=T_w, \;\;\; D_B\frac{\partial C}{\partial y} + \frac{D_T}{T_\infty }\frac{\partial T}{\partial y}=0, \;\;\; N = N_w \;\;\;\;\ at \;\;\; y =0,\nonumber \\&u \rightarrow 0, \;\;\; T\rightarrow T_\infty , \;\;\; C \rightarrow C_\infty , \;\;\; N \rightarrow N_\infty \;\;\;\;\ as \;\;\; y \rightarrow \infty \end{aligned}$$
  where *u* and *v* are velocity components in *x* and *y* directions respectively, $c_h$ is the chemotaxis constant, $W_c$ is the maximum cell swimming speed, *m* is the velocity power index, $T_f$ is the local fluid, *T* is the temperature, *C* is the nanoparticle, *N* is the density of motile micro-organisms, *p* is the pressure, $\rho _f, \rho _p, \rho _m$ are the density of nanofluid, nanoparticles and micro-organisms respectively, $D_B, D_T, D_m$ are the Brownian diffusion coefficient, thermophoresis diffusion coeffient and diffusivity of micro-organisms respectively, $R_1$ and $R_2$ are the non-Newtonian material constants, $\kappa , \sigma$ are the thermal and electrical conductivity of the fluid respectively, $\alpha$ is the thermal diffusivities, $\tau$ is the ratio of the effective heat capacitance of the nanoparticle to that of the base fluid.

For the sake of non-dimensionalization and parameterization of Eqs. (2), (3), (4) and (5) subject to boundary conditions (6) and (7), the following two sets of similarity variables are considered corresponding to the following models:

(i) In the actively controlled nanofluid model,
  8 $$\begin{aligned} \eta = \frac{a^{\frac{1}{2}}}{\vartheta ^{\frac{1}{2}}} y, \;\;\;\;\; \frac{\psi (x,y)}{x a^{\frac{1}{2}}\vartheta ^{\frac{1}{2}}}=f(\eta ),\;\;\;\;\; \theta (\eta ) =\frac{T-T_\infty }{T_w-T_\infty },\;\;\;\;\; \phi =\frac{C-C_\infty }{C_w-C_\infty },\;\;\ w(\eta ) = \frac{N-N_\infty }{N_w-N_\infty }, \end{aligned}$$
(ii) In the passively controlled nanofluid model,
  9 $$\begin{aligned} \eta = \frac{a^{\frac{1}{2}}}{\vartheta ^{\frac{1}{2}}} y, \;\;\;\;\; \frac{\psi (x,y)}{x a^{\frac{1}{2}}\vartheta ^{\frac{1}{2}}}=f(\eta ),\;\;\;\;\; \theta (\eta ) =\frac{T-T_\infty }{T_w-T_\infty },\;\;\ \phi =\frac{C-C_\infty }{C_\infty },\;\;\;\;\; w(\eta ) = \frac{N-N_\infty }{N_w-N_\infty }, \end{aligned}$$
  Introducing $\psi (x,y)$ which is the stream function, the continuity Eq. (1) is satisfied automatically and other similarity variables, Eqs. (2)–(5) become
  10 $$\begin{aligned}&\frac{d^3 f}{d \eta ^3} - \left( \frac{df}{d \eta }\right) ^2 + f \frac{d^2 f}{d \eta ^2} + K_1 \left( \frac{d^2 f}{d \eta ^2}\right) ^2 \frac{d^3 f}{d\eta ^3} + G_r\theta - N_r \phi + R_b w- M \frac{df}{d\eta }\nonumber \\&\quad + K_2 \left[ \frac{df}{d\eta }\left( \frac{d^2 f}{d \eta ^2}\right) ^2 \frac{d^3 f}{d\eta ^3} - f \frac{d^2 f}{d\eta ^2}\left( \frac{d^3 f}{d \eta ^3}\right) ^2 -f \left( \frac{d^2 f}{d\eta ^2}\right) ^2 \frac{d^4 f}{d \eta ^4} + \left( \frac{d^2 f}{d\eta ^2}\right) ^4\right] =0, \end{aligned}$$
  11 $$\begin{aligned}&\frac{d^2\theta }{d\eta ^2} + P_r f\frac{d\theta }{d\eta }-P_r\delta _t \left( f^2 \frac{d^2\theta }{d\eta ^2} + f\frac{df}{d\eta }\frac{d\theta }{d\eta }\right) + P_rN_b \frac{d\phi }{d\eta }\frac{d \theta }{d\eta } + P_r N_t \left( \frac{d \theta }{d\eta }\right) ^2 =0 \end{aligned}$$
  12 $$\begin{aligned}&\frac{d^2\phi }{d\eta ^2} + L_ef\frac{d\phi }{d\eta } -L_e\delta _n \left( f^2 \frac{d^2 \phi }{d\eta ^2} + f \frac{df}{d\eta }\frac{d\phi }{d\eta }\right) + \frac{N_t}{N_b}\frac{d^2\theta }{d\eta ^2} =0 \end{aligned}$$
  13 $$\begin{aligned}&\frac{d^2w}{\varsigma d\eta ^2} + \frac{S_{cm}}{\varsigma }f\frac{dw}{d\eta }-P_e\frac{d^2\phi }{d\eta ^2}-\frac{P_e}{\varsigma }w\frac{d^2 \phi }{d\eta ^2}-\frac{P_e}{\varsigma }\frac{d\phi }{d\eta }\frac{dw}{d\eta }=0 \end{aligned}$$
  subject to two kinds of the dimensionless boundary conditions

(i) The actively controlled nanofluid model,
  14 $$\begin{aligned}&f(0)=0,\;\;\; \frac{df(0)}{d \eta } = 1,\;\;\; \theta (0)=1, \;\;\; \phi (0)=1,\;\;\; w(0)=1, \end{aligned}$$
  15 $$\begin{aligned}&\frac{df(\infty )}{d \eta }=0,\;\;\; \theta (\infty ),\;\;\; \phi (\infty )=0,\;\;\; w(\infty )=0 \end{aligned}$$
(ii) The pasively controlled nanofluid model,
  16 $$\begin{aligned}&f(0)=0,\;\;\; \frac{df(0)}{d\eta }= 1,\;\;\; \theta (0)=1, \;\;\; N_b\frac{d\phi (0)}{d\eta }+N_t\frac{d\theta (0)}{d\eta },\;\;\; w(0)=1, \end{aligned}$$
  17 $$\begin{aligned}&\frac{df(\infty )}{d\eta }=0,\;\;\; \theta (\infty ),\;\;\; \phi (\infty )=0,\;\;\; w(\infty )=0 \end{aligned}$$

In the dimensionless equations defined above, $S_{cm}$ is the Schimdt number for diffusing motile microorganisms, $L_e$ is the Lewis number, $P_r$ is the Prandtl number, $G_r$ is the Grashof number, $N_r$ is the buoyancy-ratio parameter, $R_b$ is the bioconvection Rayleigh number, $N_b$ is the Brownian motion parameter, $N_b$ is the thermophoresis parameter, $P_e$ is bioconvection Peclet number.

In the above equations, primes denote differentiation with respect to $\eta$. The dimensionless velocity, temperature, concentration and density of microorganisms are represented as $f(\eta ), \theta (\eta ), \phi (\eta )$ and $w(\eta )$. $P_r=\frac{\vartheta }{\alpha }$ is the Prandtl number, $M=\sigma \frac{B_o^2}{\rho a}$ is the magnetic parameter, $K_1 = -\frac{6R_1a^3x^2}{\rho \vartheta ^2}$ and $K_2 = \frac{4R_2a^4x^2}{\rho \vartheta ^2}$ are the non-Newtonian parameters, $G_r = \frac{g\beta (1-C_\infty )(T_f-T_\infty )}{a^2x}$ is the modified local Grashof number, $N_r = \frac{g\phi (C_w-C_\infty )(p_\rho -p_f)}{a^2x}$ is the buoyancy-ratio parameter, $R_b =\frac{gw \gamma (C_w-C_\infty )(N-N_\infty )}{a^2x}$ is the bioconvection Rayleigh number, $N_b=\frac{\rho C)_p }{\vartheta (\rho C)_f}D_B (C_w-C_\infty )$ is the Brownian motion parameter, $N_t = \frac{\rho C)_p}{\vartheta (\rho C)_f}\frac{D_T}{T_\infty }(T_w-T_\infty )$ is the thermophoresis parameter, $\delta _t = \lambda _1a$ is the relaxation time parameter of temperature, $L_e = \frac{\vartheta }{D_B}$ is the Lewis number, $\delta _n = \lambda _2a$ is the relaxation time parameter of nanoparticle volume fraction, $\varsigma = \frac{N_\infty }{(N_w - N_\infty )}$ is gyrotactic microorganisms concentration difference parameter, $S_{cm} = \frac{\vartheta }{D_n}$ is the Schmidth number for diffusing motile microorganisms and $P_e = \frac{bW_c}{D_n}$ is the Peclet number.

The shear stress, the local heat flux, the local mass flux, and the motile microorganisms flux on the surface are $\tau _w$, $q_w$, $q_m$ and $q_n$ respectively. They are defined as;

18 $$\begin{aligned} \tau _w = \mu \left( \frac{\partial u}{\partial y}\right) _{y=0}, \;\; q_w = -k \left( \frac{\partial T}{\partial y}\right) _{y=0}, \;\; q_m = -D_B \left( \frac{\partial C}{\partial y}\right) _{y=0}, \;\; q_n = -D_m \left( \frac{\partial N}{\partial y}\right) _{y=0}. \end{aligned}$$

the local skin friction $C_f$, local Nusselt number $Nu_x$, the local Sherwood number $Sh_x$ and local density number of motile microorganism $Nn_x$ are defined as;

19 $$\begin{aligned} C_f = \frac{\tau _w}{\rho u^2_w}, \;\;Nu_x = \;\; \frac{xq_w}{\kappa (T_w-T_\infty )},\;\;Sh_x = \frac{xq_m}{D_B(C_w-C_\infty )},\;\; Nu_x = \frac{xq_n}{D_m(N_w-N_\infty )} \end{aligned}$$

In dimensional practice these quantities can be written as

20 $$\begin{aligned} \sqrt{Re_x}C_f = -f''(0), \;\; \frac{Nu_x}{\sqrt{Re_x}} = -\theta '(0),\;\; \frac{Nn_x}{\sqrt{Re_x}} = -w'(0), \end{aligned}$$

where $Re_x=\frac{u_wx}{\vartheta }$ denotes local Reynolds number.

### Optimal homotopy analysis solutions

In many cases, by means of analyzing the physical background and the initial/boundary conditions of the nonlinear differential problem, we might know what kinds of base functions are proper to represent the solution, even without solving the given nonlinear problem. In view of the boundary conditions (18)–(21), $f(\eta )$, $\theta (\eta )$, $\phi (\eta )$ and $w(\eta )$ can be expressed by the set of base functions in the form

21 $$\begin{aligned} \langle \eta ^{j}exp(-nj) \mid j\ge 0, n \ge 0 \rangle \end{aligned}$$

The solutions $f(\eta )$ and $\theta (\eta )$ can be represented in a series form as

22 $$\begin{aligned}&f(\eta ) = a_{0, 0}^{0} +\sum _{n=0}^{\infty }\sum _{k=0}^{\infty } a_{n, k}^{k} \eta ^{k} exp(-nj) \end{aligned}$$

23 $$\begin{aligned}&\theta (\eta ) = \sum _{n=0}^{\infty }\sum _{k=0}^{\infty } b_{n, k}^{k} \eta ^{k} exp(-nj) \end{aligned}$$

24 $$\begin{aligned}&\phi (\eta ) = \sum _{n=0}^{\infty }\sum _{k=0}^{\infty } c_{n, k}^{k} \eta ^{k} exp(-nj) \end{aligned}$$

25 $$\begin{aligned}&w(\eta ) = \sum _{n=0}^{\infty }\sum _{k=0}^{\infty } d_{n, k}^{k} \eta ^{k} exp(-nj) \end{aligned}$$

In which $a_{n, k}^{k}$, $b_{n, k}^{k}$, $c_{n,k}^{k}$ and $d_{n,k}^{k}$ are the coefficients. As long as such a set of base functions are determined, the auxiliary function $H(\eta )$, the initial approximation $f_{o} (\eta )$, $\theta _{o} (\eta )$, $\phi _{o}(\eta )$ and $w_o(\eta )$ and the auxiliary linear operator $L_{f}, L_{\theta }, L_{\phi }$ and $L_{w}$ must be chosen in such a way that all solutions exist and can be expressed by these sets of base functions. Therefore, in framing the Homotopy Analysis Method (HAM), we apply the rule of solution expressions in choosing the auxilliary function $H(\eta )$, initial approximation $f_o(\eta ), \theta _o(\eta ), \phi _o(\eta )$ and $w_o(\eta )$.

Invoking the rule of solution expressions for $f(\eta ), \theta (\eta ), \phi (\eta )$ and $w(\eta )$ for Eqs. (14)–(17) together with (18)–(21) are expressed as

For actively controlled nanofluid model

26 $$\begin{aligned} f_{o}(\eta ) = 1 - exp(-\eta ),  \theta _{o}(\eta ) = exp(-\eta ), \phi _o(\eta )= exp(-\eta ), w_o(\eta )= exp(-\eta ) \end{aligned}$$

For passively controlled nanofluid model

27 $$\begin{aligned} f_{o}(\eta ) = 1 - exp(-\eta ),  \theta _{o}(\eta ) = exp(-\eta ),  \phi _o(\eta )=-\frac{N_t}{N_b}exp(-\eta ), w_o(\eta )=exp(-\eta ) \end{aligned}$$

with linear operators $L_{f}, L_{\theta }, L_{\phi }$ and $L_{w}$ as

28 $$\begin{aligned}&L_{f}[f(\eta ; q)] = \frac{\partial ^{3} f(\eta ; q)}{\partial \eta ^{3}} - \frac{\partial f(\eta ; q)}{\partial \eta } \end{aligned}$$

29 $$\begin{aligned}&L_{\theta }[\theta (\eta ; q)] = \frac{\partial ^{2} \theta (\eta ; q)}{\partial \eta ^{2}} - \theta (\eta ; q) \end{aligned}$$

30 $$\begin{aligned}&L_{\phi }[\phi (\eta ; q)] = \frac{\partial ^{2} \phi (\eta ; q)}{\partial \eta ^{2}} - \phi (\eta ; q) \end{aligned}$$

31 $$\begin{aligned}&L_{w}[w(\eta ; q)] = \frac{\partial ^{2} w(\eta ; q)}{\partial \eta ^{2}} - w(\eta ; q) \end{aligned}$$

The linear operators $L_{f}, L_{\theta }, L_{\phi }$ and $L_{w}$ have the following properties

32 $$\begin{aligned}&L_{f}[C_{1} + C_{2}exp(-\eta ) +C_{3}exp(\eta )] = 0, \quad L_{\theta }[C_{4}exp(-\eta ) + C_{5}] = 0,\nonumber \\&L_{\phi }[C_{6}exp(-\eta ) + C_{7}] = 0,\quad L_{w}[C_{8}exp(-\eta ) + C_{9}] = 0 \end{aligned}$$

In which $C_{1}$, $C_{2}$, $C_{3}$, $C_{4}$, $C_{5}, C_{6}, C_{7}, C_{8}$, and $C_{9}$ are constants. The linear operators were solved using Wolfram Mathematica to obtain the vales of skin friction coefficient $f''(0)$, Nusselt number $-\theta '(0)$, Sherwood number $-\phi '(0)$ and density number $-w'(0)$ for various values of thermophoresis parameter $N_t$

## Results and discussion

The flow of thixotropic-nanofluid in a water-based suspension has been computed for various values of emerging parameters. On velocity profiles $f'(\eta )$, temperature profiles $\theta (\eta )$, nanoparticles concentration profiles $\phi (\eta )$, and density of motile microorganisms profiles $w(\eta )$, the effects of certain selected parameters have been evaluated and presented. The theoretical values of the governing parameters for both cases of actively controlled nanofluid model and passively controlled nanofluid model are $M=G_r=N_r=R_b=0.5, N_b=N_t=\varsigma =\delta _t=\delta _n=K_1=K_2=0.1, S_c=P_e=1.0$ and $P_r=L_e=2.0$. The variations of local Nusselt numbers for both cases of passive and active controls of nanoparticles are depicted in Tables 1 and 2. In Table 1 it is revealed that the local Nusselt number is a decreasing function of $P_r, N_b$ and $N_t$ for the case of passive control of nanoparticles. Likewise, in Table 2 it is noticed that the local Nusselt number is a decreasing function of $P_r, N_b$ and $N_t$ for active control of nanoparticles.

**Table 1:** Numerical values of $\frac{Nu_x}{\sqrt{Re_x}}=-\theta '(0)$ for various values of $P_r$, $N_b$ and $N_t$ when $M = G_r = N_r=R_b=0.5, P_e =2.0, \varsigma = \delta _n = \delta _t =K_1 =K_2=0.1.$

$P_r$	$N_b$	$N_t$	$\frac{Nu_x}{\sqrt{Re_x}}=-\theta '(0)$
0.1	0.1	0.1	1.3692
0.7	0.3	0.3	1.2696
1.4	0.5	0.5	0.5185

**Table 2:** Numerical values of $\frac{Nu_x}{\sqrt{Re_x}}=-\theta '(0)$ for various values of $P_r$, $N_b$ and $N_t$ when $M = G_r = N_r=R_b=0.5, P_e =2.0, \varsigma = \delta _n = \delta _t =K_1 =K_2=0.1.$

$P_r$	$N_b$	$N_t$	$\frac{Nu_x}{\sqrt{Re_x}}=-\theta '(0)$
0.1	0.1	0.1	1.2932
0.7	0.3	0.3	0.8932
1.4	0.5	0.5	0.6189

**Figure 1:**
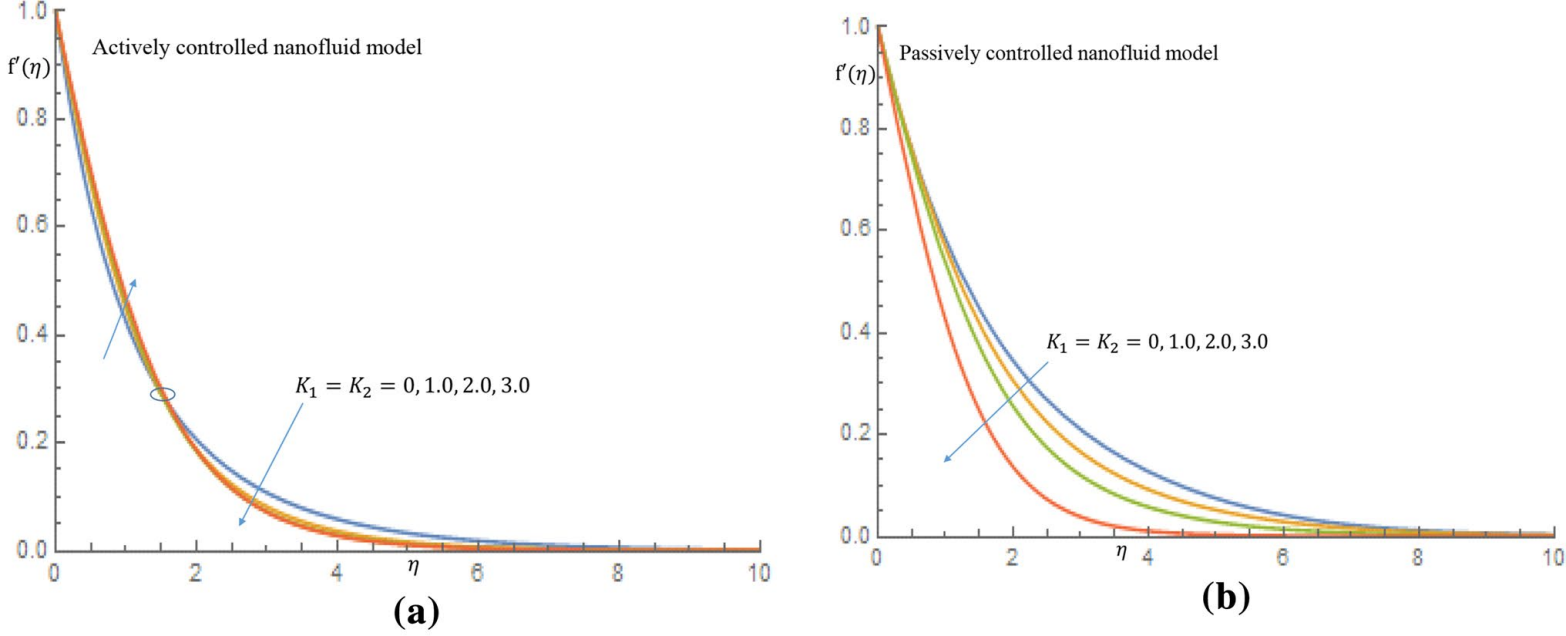
(**a**) Effect of $K_1=K_2$ on velocity profile when $\delta _t=\delta _n=0.1$. (**b**) Effect of $K_1=K_2$ on velocity profile when $\delta _t=\delta _n=0.1$.

The velocity of the thixotropic-nanofluid increases slightly in the event of active control of nanoparticles as seen in Fig. 1a. When $\eta =1.68$, all profiles merged and the fluid velocity dropped towards the free stream.

**Figure 2:**
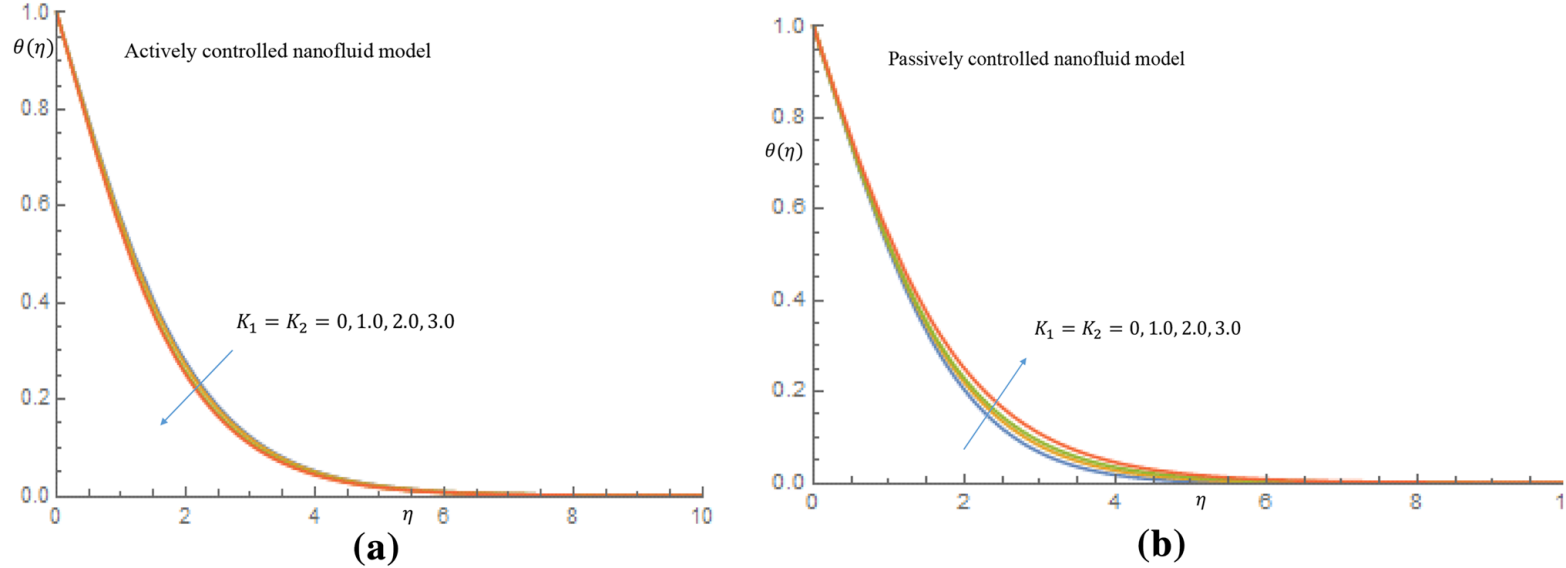
(**a**) Effect of $K_1=K_2$ on temperature profile when $\delta _t=\delta _n=0.1$. (**b**) Effect of $K_1=K_2$ on temperature profile when $\delta _t=\delta _n=0.1$.

**Figure 3:**
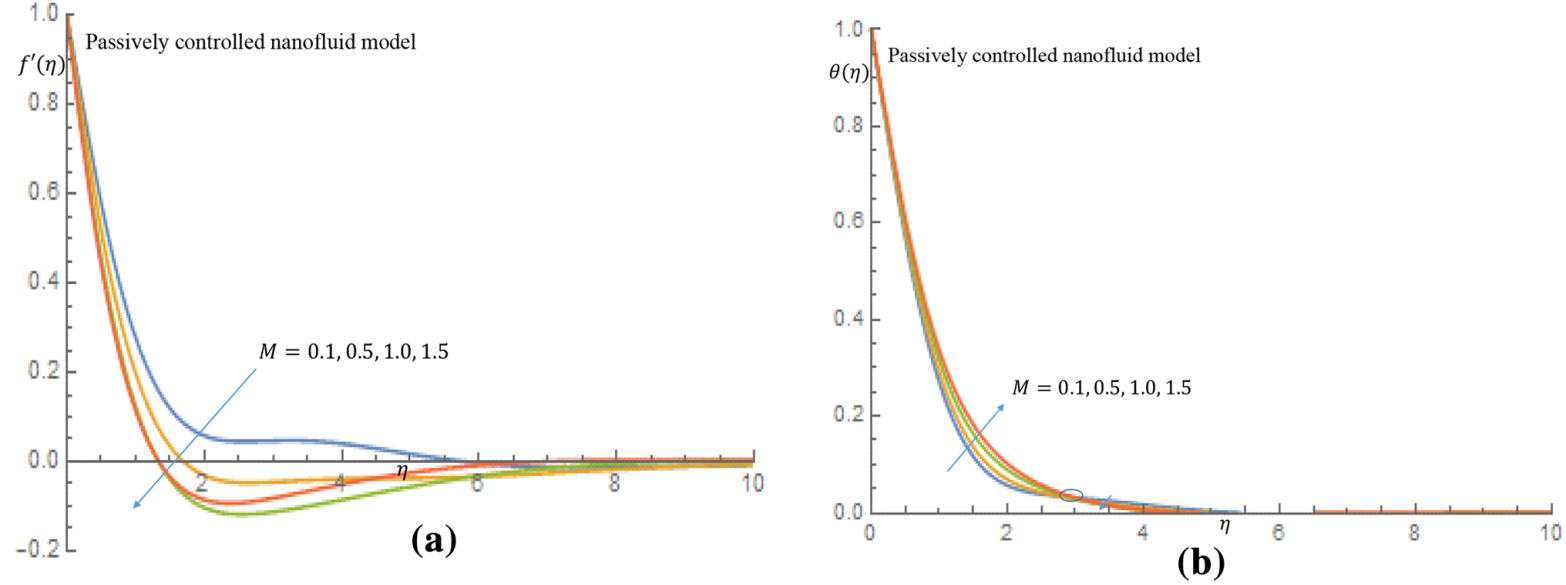
(**a**) Effect of *M* on velocity profile when $G_r=1.0$. (**b**) Effect of *M* on temperature profile when $G_r=1.0$.

In Fig. 1b for case of passive control of nanoparticles, it is revealed that as thixotropic parameters increase, there is a noticeble decrease in the velocity of the fluid. Figure 2a shows that the temperature of the fluid decreases as the thixotropic parameters $K_1, K_2$ increase in the case of active control of nanoparticles, while in Fig. 2b increamental values of $K_1, K_2$ leads to rise in the temperature of the thixotropic-nanofluid in case of passive control of nanoparticles. In Fig. 3a it is observed that the velocity distribution declines with the incremental values of magnetic parameter *M*, while in Fig. 3b, the temperature distribution is enhanced as *M* increases. Physically, the introduction of magnetic field to the flow directions induces a Lorentz force, which has the ability to retard the flow of fluid and thereby reduce the velocity of fluid and enhance the temperature of fluid.

**Figure 4:**
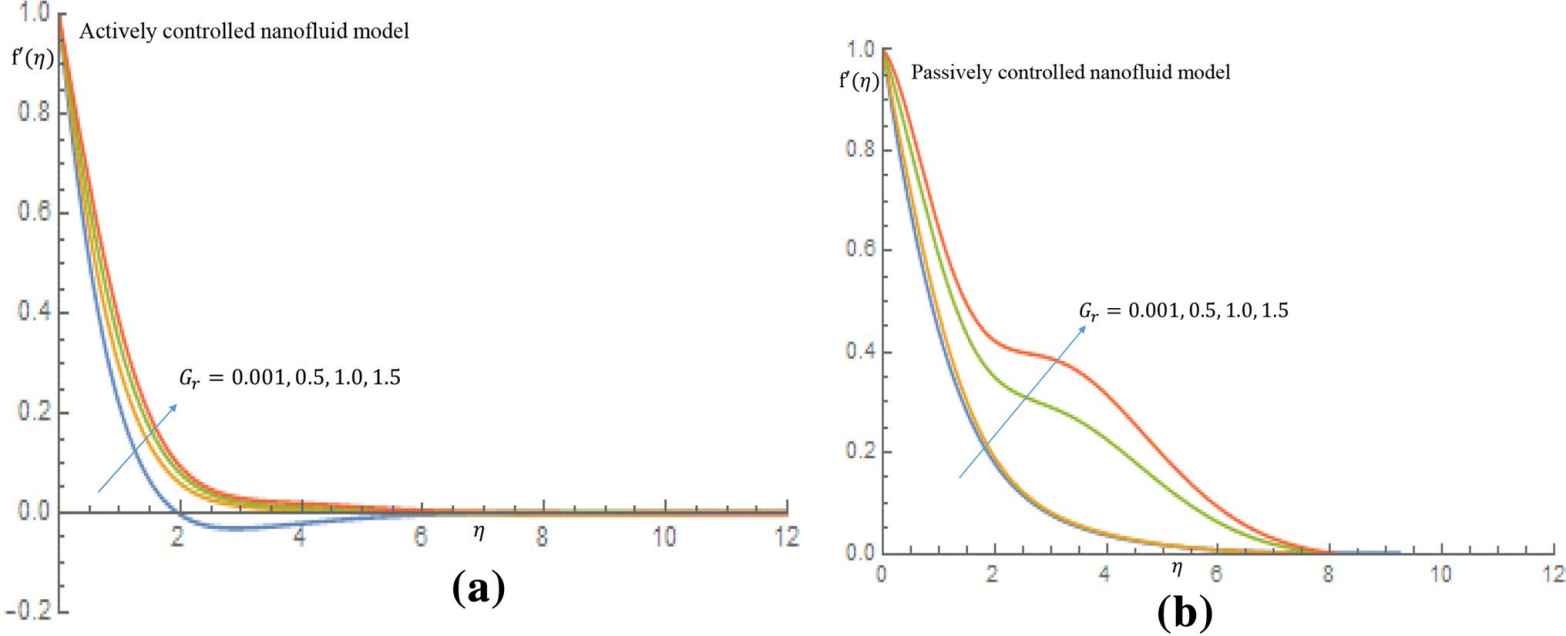
(**a**) Effect of $G_r$ on velocity profile when $N_r=N_b=0.75$. (**b**) Effect of $G_r$ on velocity profile when $N_r=N_b=0.75$.

**Figure 5:**
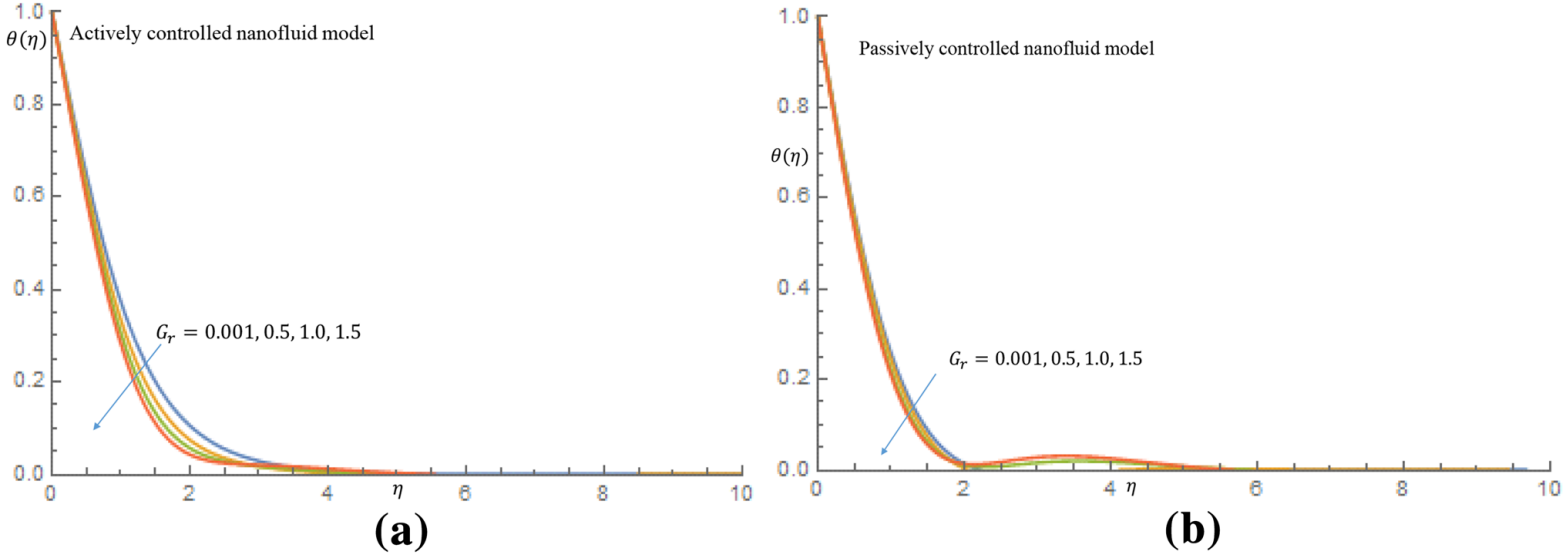
(**a**) Effect of $G_r$ on temperature profile when $N_r=N_b=0.75$. (**b**) Effect of $G_r$ on temperature profile when $N_r=N_b=0.75$.

**Figure 6:**
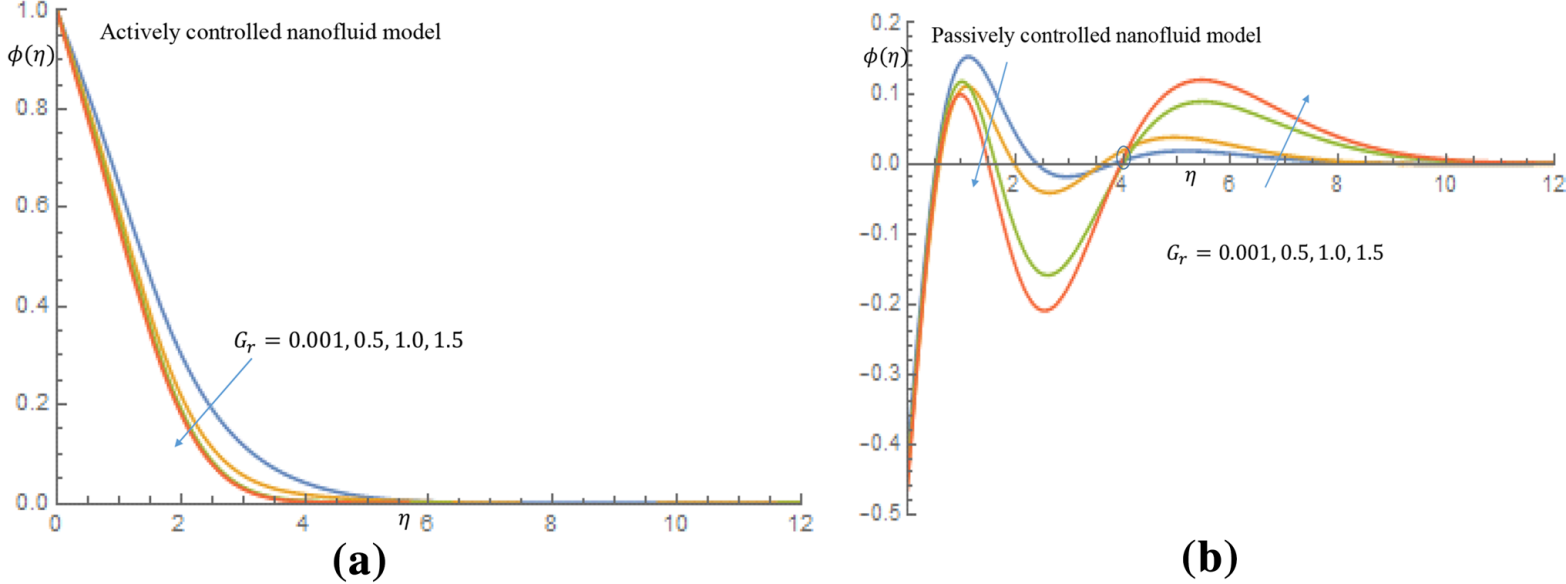
(**a**) Effect of $G_r$ on velocity profile when $N_r=N_b=0.75$. (**b**) Effect of $G_r$ on velocity profile when $N_r=N_b=0.75$.

In Fig. 4a it is noticed that as the $G_r$ increases from 0.001 through 0.5, 1.0 to 1.5 for the case of active control of nanoparticles the velocity distribution slightly increases and decay towards $\eta =\infty$ and also in case of passive control of nanoparticles, there is rise in the velocity profile, as seen in Fig. 4b. The observed fluid velocity behavior is owing to the fact that gravitational force increases mobility on the vertical surface. It is revealed in Fig. 5a in case of active control of nanoparticles that as $G_r$ increases temperature profile decreases within the domain $0.67 \le \eta \le 2.50$ at $\eta =2.52$ all profiles merged together and decay towards $\eta =\infty$, where as in Fig. 5b when nanoparticles are controlled passively, the temperature profiles decrease slightly, and all profiles fused together at $\eta =2.0$, and later decay towards the free stream. It is observed from Fig. 6a that as $G_r$ increases for the cases of active control of nanoparticles, concentration profile decreases along the wall. It is noticed from Fig. 6b for case of passive control of nanoparticles that as $G_r$ is increased all profiles merged at a distance very close to the wall. It is later observed that at $\eta =0.58$ the concentration of nanoparticles decline away from wall within the domain $0.58 \le \eta \le 3.75$ and later on, the profiles merged together at $\eta =4.23$ and thereafter, there is a substantial enhancement in the nanoparticles concentration. It is obvious that there is a noticeable difference in the behaviour of concentration of nanoparticles suspended in highly viscous thixotropic fluid for both cases of nanofluid model in consideration.

**Figure 7:**
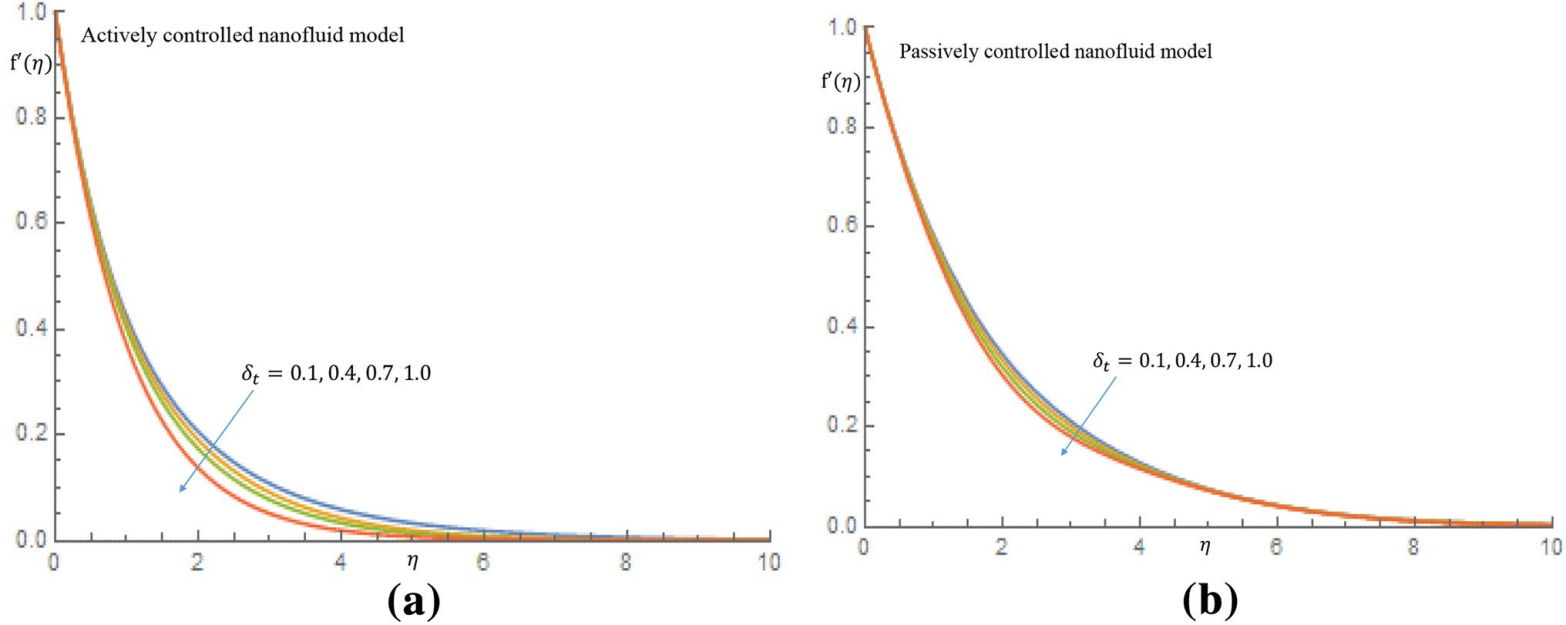
(**a**) Effect of $\delta _t$ on velocity profile when $K_1=K_2=0$. (**b**) Effect of $\delta _t$ on velocity profile when $K_1=K_2=0$.

**Figure 8:**
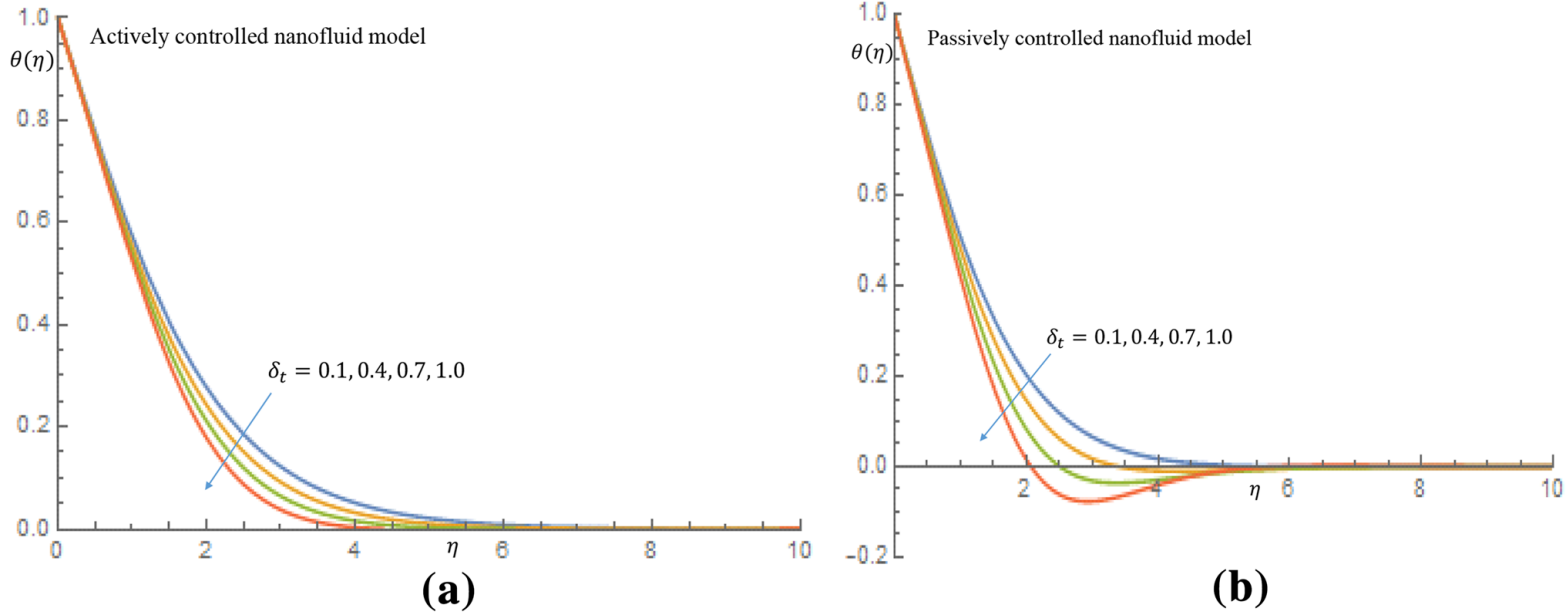
(**a**) Effect of $\delta _t$ on temperature profile when $K_1=K_2=0$. (**b**) Effect of $\delta _t$ on temperature profile when $K_1=K_2=0.$.

**Figure 9:**
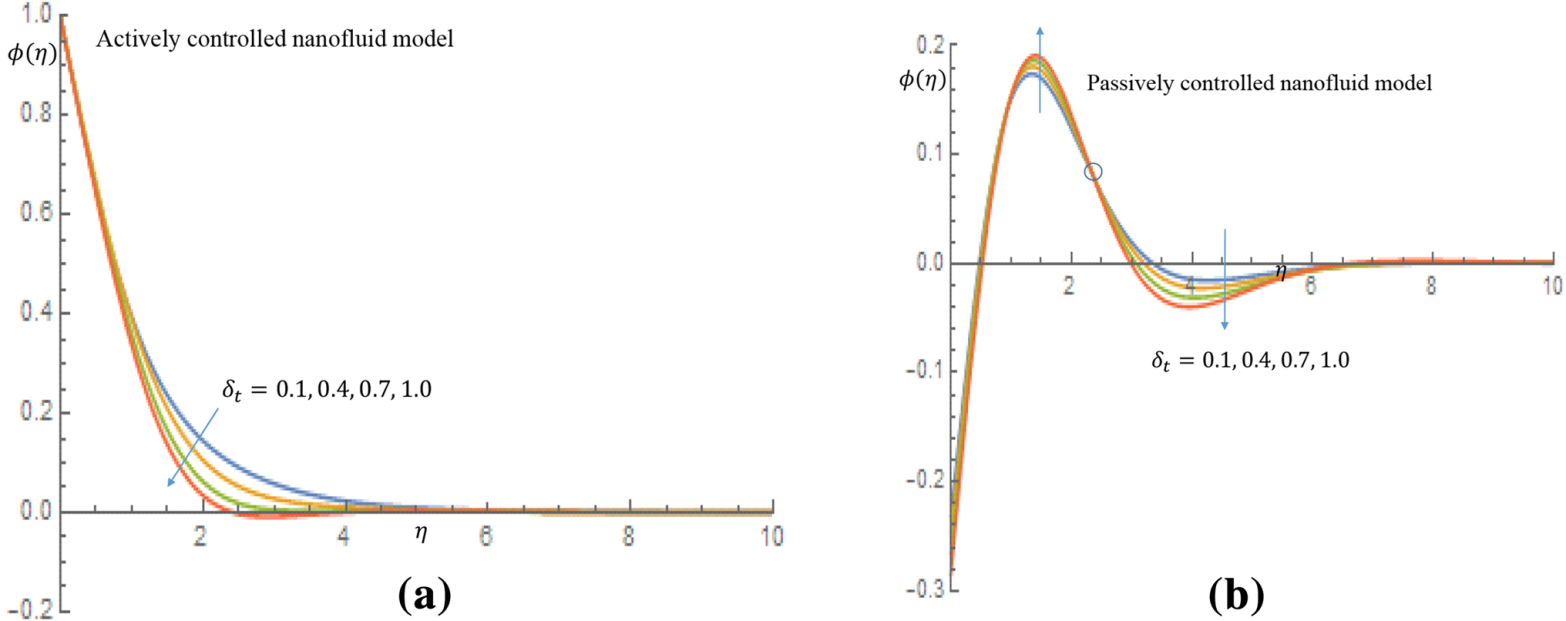
(**a**) Effect of $\delta _t$ on nanoparticles concentration profile when $K_1=K_2=0$. (**b**) Effect of $\delta _t$ on nanoparticles concentration profile when $K_1=K_2=0$.

**Figure 10:**
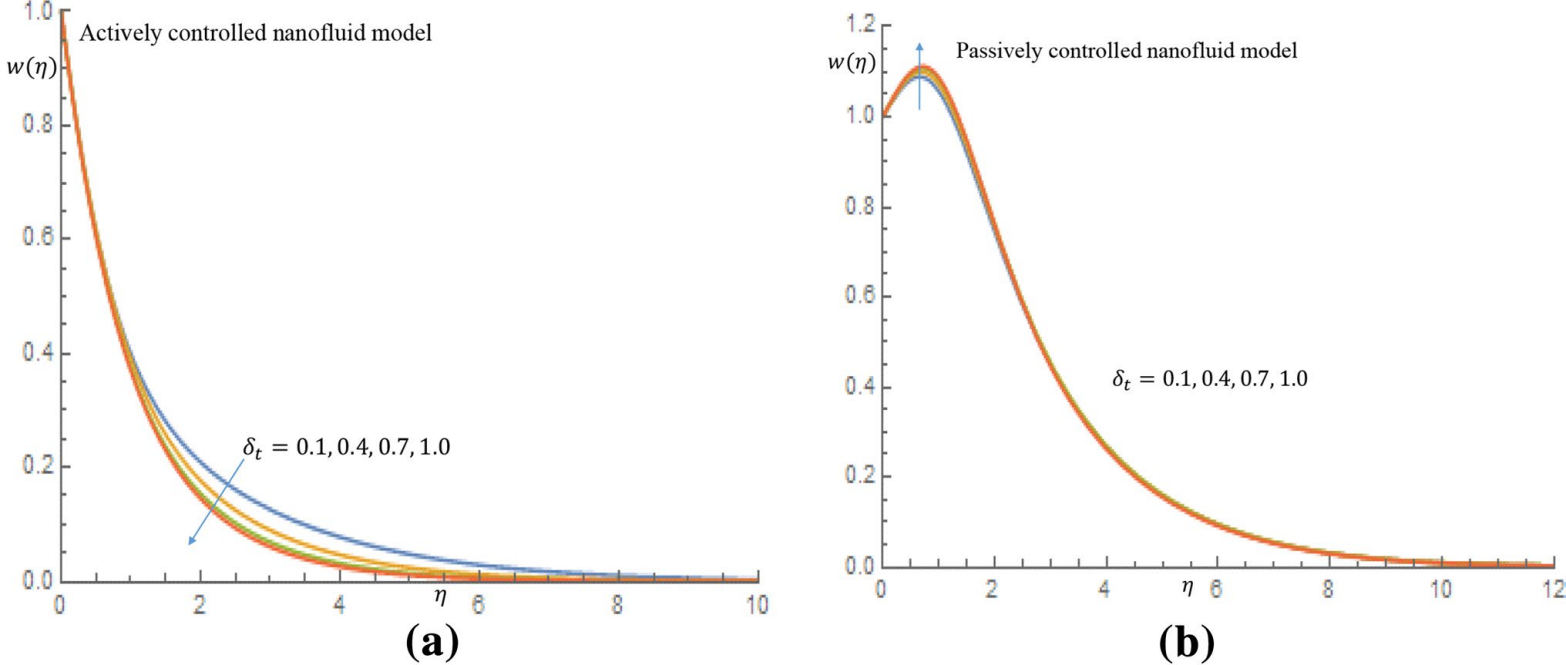
(**a**) Effect of $\delta _t$ on density of motile microorganisms profile when $K_1=K_2=0$. (**b**) Effect of $\delta _t$ on density of motile microorganisms profile when $K_1=K_2=0$.

**Figure 11:**
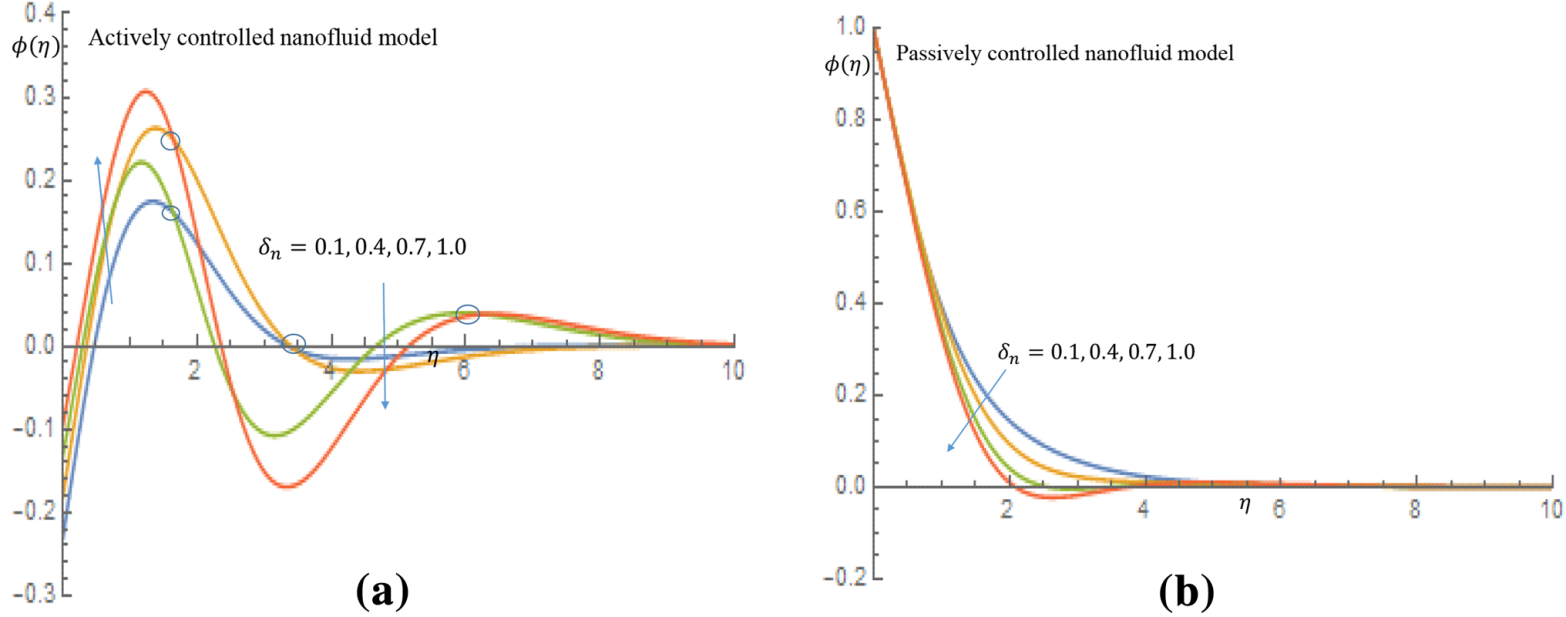
(**a**) Effect of $\delta _n$ on nanoparticles concentration profile when $K_1=K_2=1.2$. (**b**) Effect of $\delta _n$ on nanoparticles concentration profile when $K_1=K_2=1.2$.

Figures 7a, 8, 9, 10b depict the influence of the thermal relaxation time parameter $\delta _t$ for $K_1=K_2=0$, which corresponds to Newtonian case. Figure 7a shows that $\delta _t$ has a diminishing influence on the velocity profile, and Fig. 7b shows a similar pattern for passive control of nanoparticles. It is shown in Fig. 8a for case of active control of nanoparticles that as $\delta _t$ increases both fluid temperature and thermal boundary layer thickness demonstrate a decelerating practice while similar behaviour is observed in Fig. 8b in the event of passive control of nanoparticles. Physically, due to thermal relaxation augmentation, material particles require more time for heat transfer to their neighboring particles. Figure 9a shows that for case of active control of nanoparticles, as $\delta _t$ increases from 0.1 through 0.4, 0.7 to 1.0, a decreasing trend in the nanoparticles concentration profile with the solutal boundary layer thickness is observed. Whereas in case of passive control of nanoparticles, a mixed trend is observed in nanoparticles concentration profile for incremental values of $\delta _t$. Physically, relaxation time parameter $\delta _t$ permits sufficient time to nanoparticles to dilute in base fluid which leads to an increasing trend with the passage of time. In Fig. 10a it is noticed that density of motile microorganisms profile decreases with increasing values of $\delta _t$ for case of actively controlled nanofluid model. Physically, this corresponds to the fact that gyrotactic cells swim back to bottom layer of the fluid resulting in reduction in the density of the motile microorganisms. Figure 10b on the other hand shows how incremental values $\delta _t$ result in a relatively slight increase in motile microorganisms density distribution in case of passive control of nanoparticles. Because gyrotactic microorganisms are considerably denser than the fluid, it takes a short time for them to swim to the top layer of the fluid causing instability in the surface of the fluid. When $K_1=K_2=1.2$, which implies a non-Newtonian thixotropic fluid, impact of $\delta _n$ on nanoparticles concentration distributions is shown in Fig. 11a,b. It is noticed in Fig. 11a that a mixed trend is observed for actively controlled model, it is further observed that concentration of nanoparticles is enhanced at the wall. For higher value of $\delta _n$ concentration of nanoparticles rapidly increases to a peak value (which is more than 0.3). In case of passive control of nanoparticles, incremental values of $\delta _n$ result in a decline in concentration profiles of nanoparticles, as shown in Fig. 11b.

**Figure 12:**
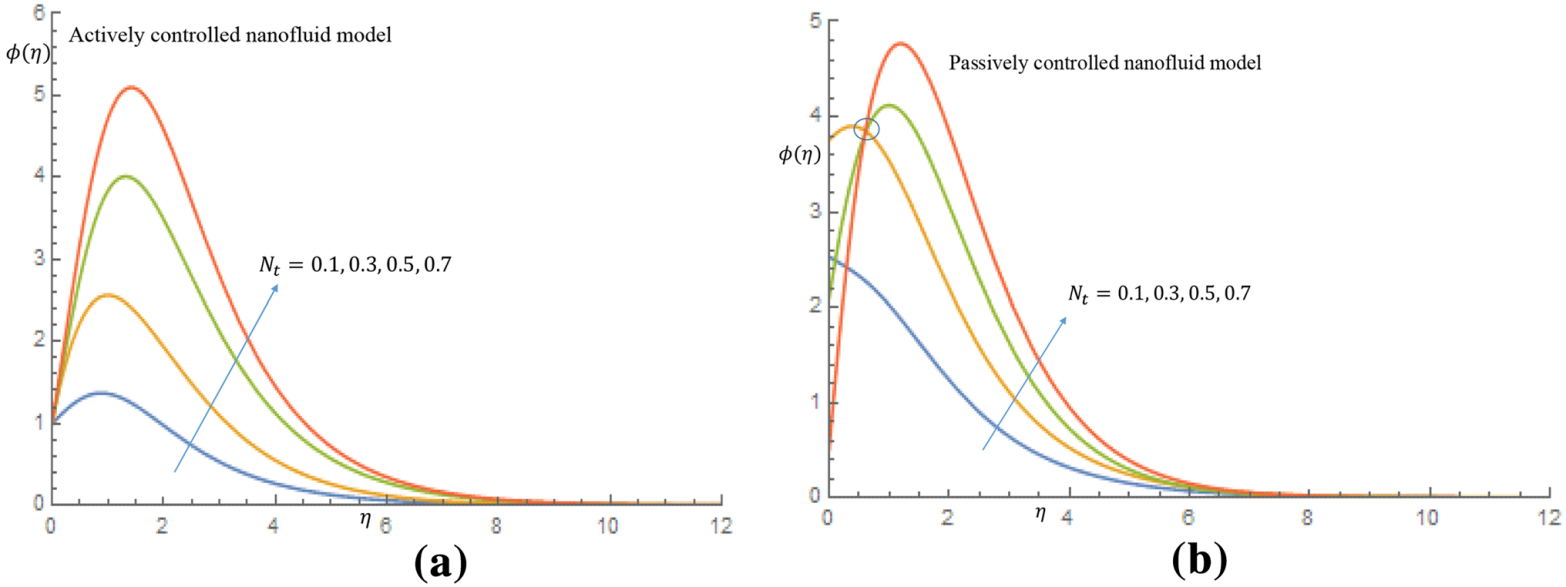
(**a**) Effect of $N_t$ on nanoparticles concentration profile when $N_b=0.05$. (**b**) Effect of $N_t$ on nanoparticles concentration profile when $N_b=0.05$.

**Figure 13:**
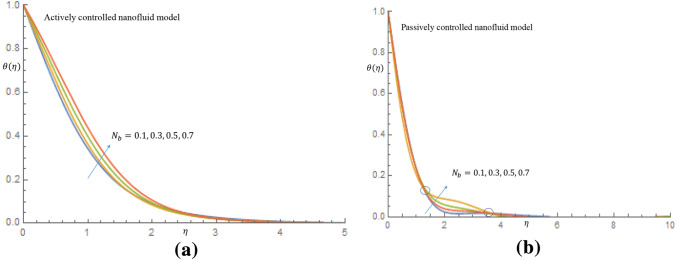
(**a**) Effect of $N_b$ on temperature profile when $N_t=0.1$. (**b**) Effect of $N_b$ on temperature profile when $N_t=0.1$.

Figure 12a manifests that by varing the $N_t$, non-dimensional nanoparticles concentration profile increases to peak value of 5.2 for case of actively controlled model. Likewise, it is revealed in Fig. 12b for case of passively controlled model that, as $N_t$ varies, the non-dimensional concentration profile increases to peak value of 4.8 and decay smoothly to the free stream. This observation is due to the fact that higher $N_t$ is expected to allow a deeper penetration of the concentration. Figure 13a,b communicate the implications of $N_b$ on the dimensionless temperature profiles respectively. Brownian motion $N_b$ can be described as the ratio of the nanoparticle diffusion to the nanofluid thermal diffusion Mahdy^26^. It is seen from Fig. 13a for case of the active control of nanoparticles that incremental values $N_b$ permits rise in temperature distribution and thermal boundary layer thickness. Physically, this is because presence of Brownian motion makes particles to be more closer together, allowing heat to be transferred between them. Also, in Fig. 13b, it is observed that all particles merged together as a result of the strong intermolecular force binding the molecules of particles suspended in thixotropic-nanofluid. Beyond this reaction, all particles diverge and the temperature profile increases between the region $1.6 \le \eta \le 3.5$, owing to presence of Brownian motion which is motivated to warm the molecules of the particles and enhances the thermal conductivity, thereby causing the temperature of the fluid to be elevated. Brownian motion contributes to efficiency of heat transfer in thixotropic-nanofluids, as evidenced by the same behavior found in these two models.

**Figure 14:**
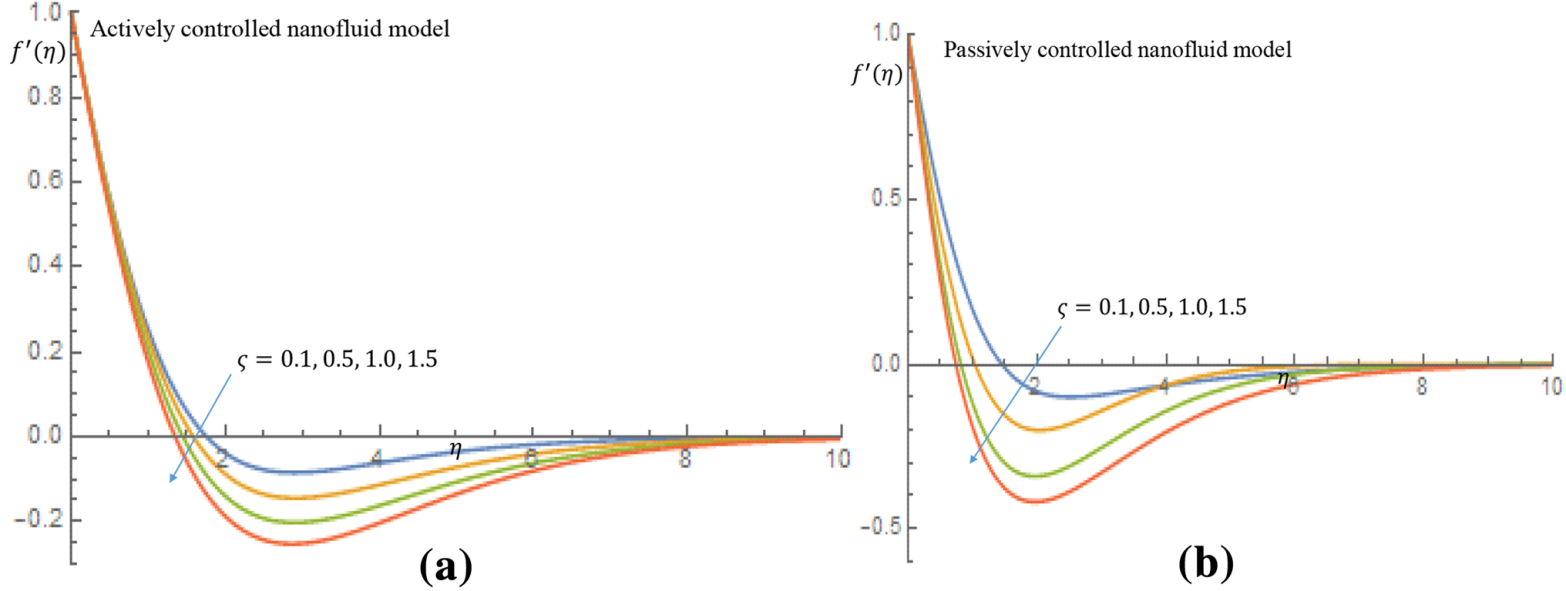
(**a**) Effect of $\varsigma$ on velocity profile when $P_e=2.0$. (**b**) Effect of $\varsigma$ on velocity profile when $P_e=2.0$.

Increasing values of $\varsigma$ correlate to reduction of the dimensionless velocity distributions for both active and passive control of nanoparticles, as shown in Fig. 14a,b. This is because density difference between the gyrotactic microorganisms and base fluid makes the surface of the fluid to become unstable as a result of cell agglomeration, causing the motile microorganisms to swim back to bottom layer of the fluid.

**Figure 15:**
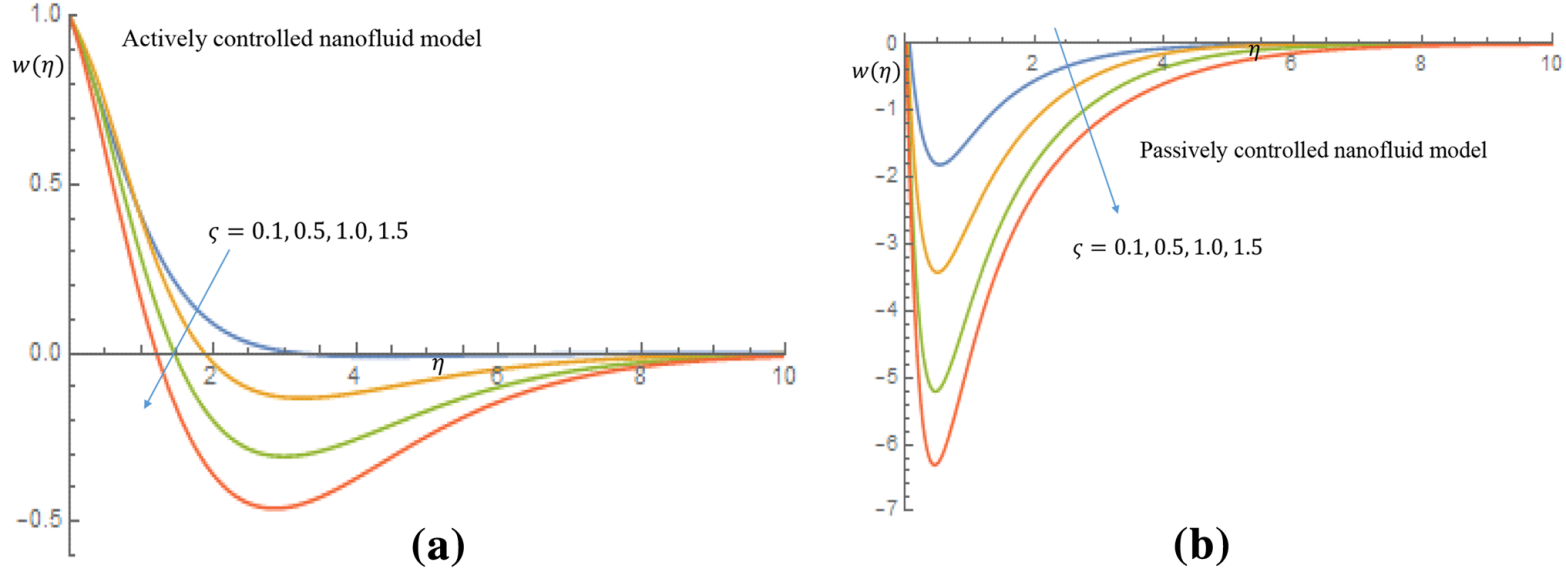
(**a**) Effect of $\varsigma$ on density of motile microorganisms profile when $P_e=2.0$. (**b**) Effect of $\varsigma$ on density of motile microorganisms profile when $P_e=2.0$.

In case of active control of nanoparticles, an increase in $\varsigma$ causes decline in density of motile microorganisms profile, as shown in Fig. 15a. Similarly, in Fig. 15b in case of passive control of nanoparticles, it is revealed that density of motile microorganisms profile decreases near the wall with peak value estimated to be 6.29 for stronger $\varsigma .$

**Figure 16:**
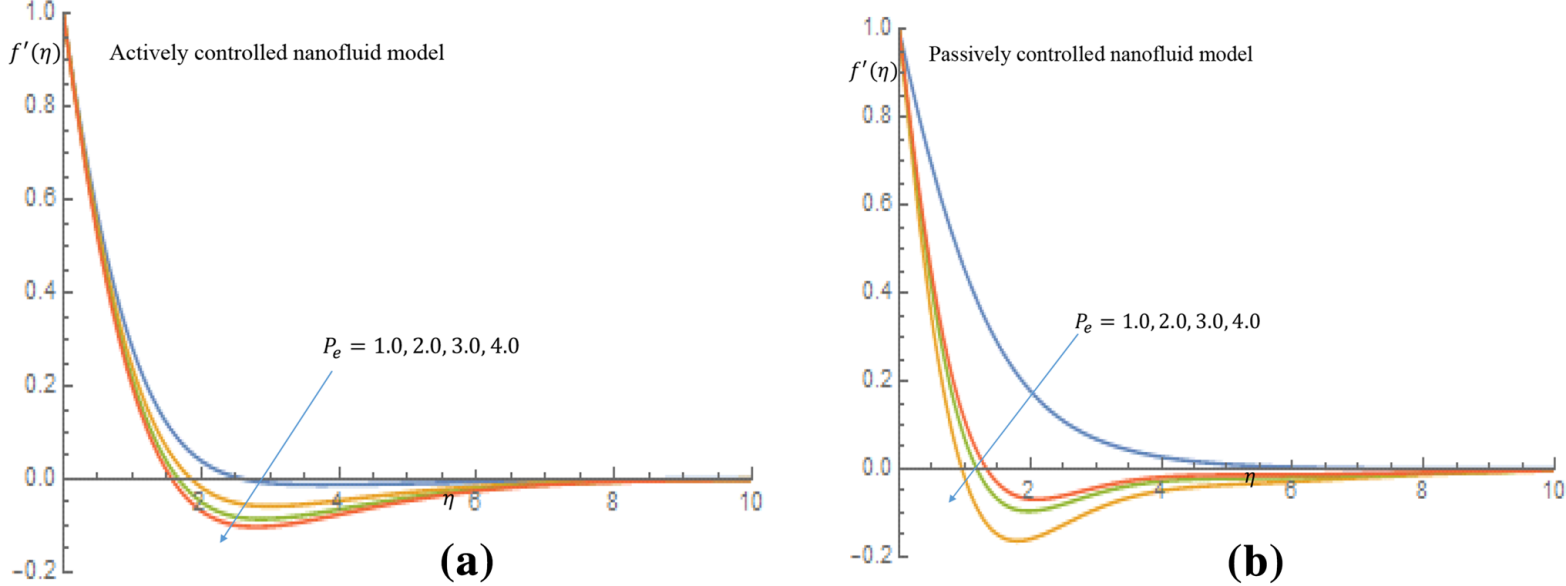
(**a**) Effect of $P_e$ on velocity profile when $L_e=2.0$. (**b**) Effect of $P_e$ on velocity profile when $L_e=2.0$.

**Figure 17:**
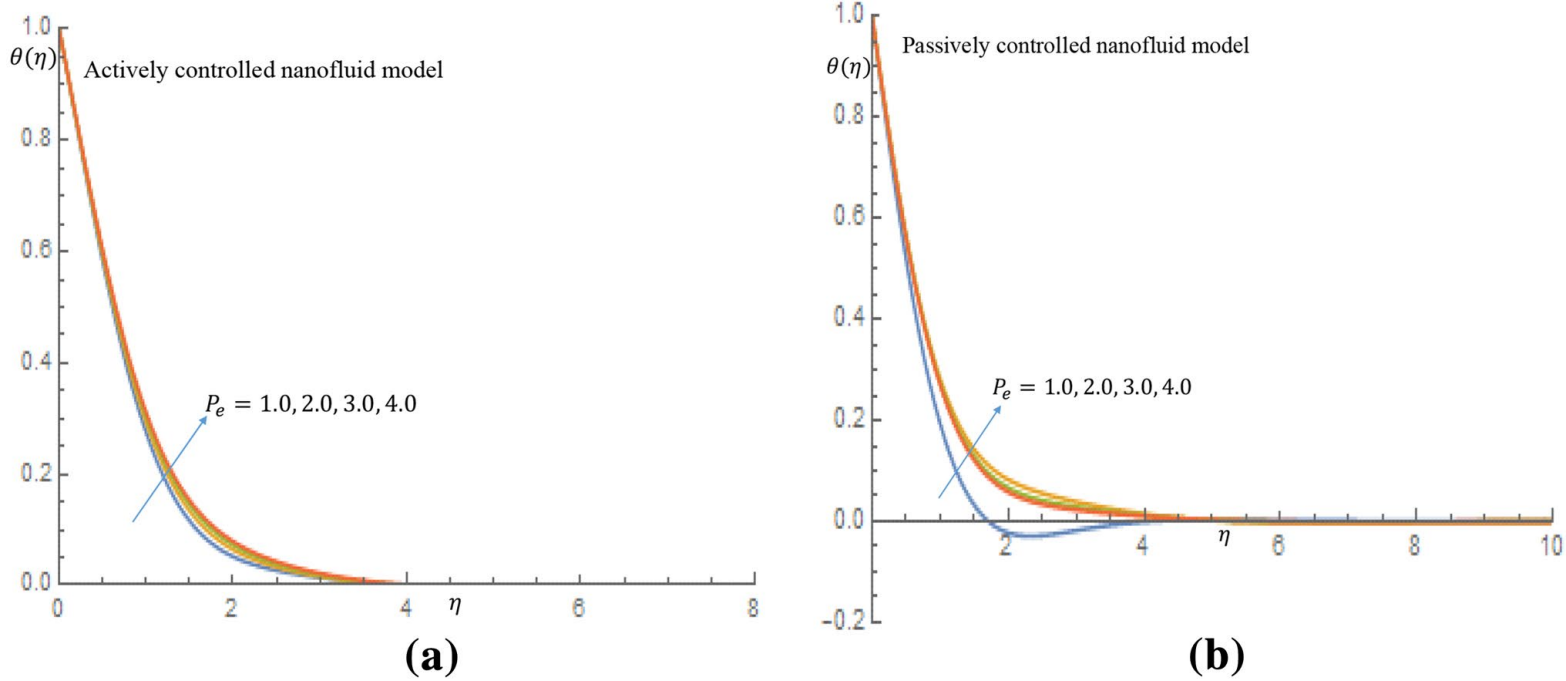
(**a**) Effect of $P_e$ on temperature profile when $L_e=2.0$. (**b**) Effect of $P_e$ on temperature profile when $L_e=2.0$.

**Figure 18:**
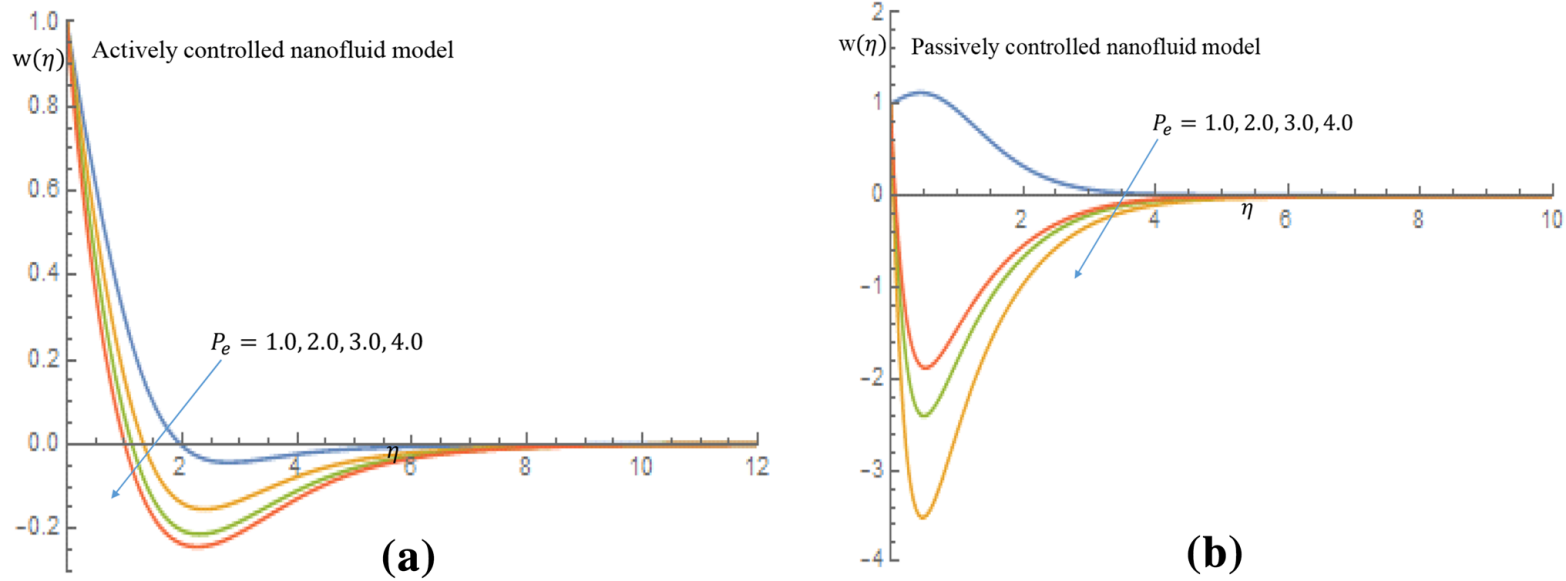
(**a**) Effect of $P_e$ on density of motile microorganisms profile when $L_e=2.0$. (**b**) Effect of $P_e$ on density of motile microorganisms profile when $L_e=2.0$.

Figure 16a,b show that for both active and passive controls of nanoparticles, fluid velocity and momentum boundary layer thickness show a decelerating trend as $P_e$ increases. It shown in Fig. 17a that increase in the values of $P_e$ result in a slight increase in temperature of the fluid for active control of nanoparticles, whereas in case of passive control of nanoparticles, there is noticeable increase in temperature of the fluid and thermal boundary layer thickness, as shown in Fig. 17b. It is clearly observed in Fig. 18a in case of active control of nanoparticles that, incremental values of $P_e$ reflect decline nature of density of motile microorganisms distribution. It is observed in Fig. 18b for the passive control of nanoparticles that increasing values of $P_e$ is a dimensionless group that represents the ratio of heat transfer via fluid motion to heat transfer by thermal conduction; this is the major reason why the temperature field increases while density of the motile microorganisms field decreases when $P_e$ value changes.

## Conclusion

The Catteneo-Christov double-diffusion model is used to investigate the features of boundary layer flow of a water-based thixotropic-nanofluid containing gyrotactic microorganisms past a vertical surface. In the study, both actively and passively controlled nanofluid models were used. To convert the governing partial differential equations into a system of ordinary differential equations a set of similarity variables have been introduced. This investigation yielded the following significant findings

1. For both active and passive control of nanoparticles, dimensionless velocities and temperatures are decreasing functions of thermal relaxation $\delta _t$. Similarly, in case of active control of nanoparticles, dimensionless concentration nanoparticle is a decreasing function of relaxation parameter $\delta _t$, but in case of passive control of nanoparticles, a mixed trend is observed.
2. Increasing solutal parameter $\delta _n$ reduces concentration of nanoparticles profiles in case of passive control of nanoparticles while a mixed trend is observed in case of active control of nanoparticles
3. Incremental values of thixotropic parameters $K_1,K_2$ correspond to increase in dimensionless fluid velocity for both active and passive control of nanoparticles
4. Increasing magnetic parameter *M* reduces velocity of the fluid and increases temperature of the fluid in case of passive control of nanoparticles
5. Incremental values of thermophoretic parameter $N_t$ leads to reduction in non-dimensional velocity profiles for both cases of active and passive controls of nanoparticles. Inside thermal boundary layer, dimensionless temperature increases with thermophoretic parameter $N_t$ for both cases of active and passive controls of nanoparticles
6. Inside the thermal boundary layer, dimensionless temperature profiles increase with Brownian motion parameter $N_b$ for both cases of active and passive controls of nanoparticles.
